# Simple generalisation of a mesophyll resistance model for various intracellular arrangements of chloroplasts and mitochondria in C_3_ leaves

**DOI:** 10.1007/s11120-017-0340-8

**Published:** 2017-02-14

**Authors:** Xinyou Yin, Paul C. Struik

**Affiliations:** Centre for Crop Systems Analysis, Wageningen University & Research, P.O. Box 430, 6700 AK Wageningen, The Netherlands

**Keywords:** CO_2_ transfer, Internal conductance, Mesophyll resistance

## Abstract

The classical definition of mesophyll conductance (*g*_m_) represents an apparent parameter (*g*_m,app_) as it places (photo)respired CO_2_ at the same compartment where the carboxylation by Rubisco takes place. Recently, Tholen and co-workers developed a framework, in which *g*_m_ better describes a physical diffusional parameter (*g*_m,dif_). They partitioned mesophyll resistance (*r*_m,dif_ = 1/*g*_m,dif_) into two components, cell wall and plasmalemma resistance (*r*_wp_) and chloroplast resistance (*r*_ch_), and showed that *g*_m,app_ is sensitive to the ratio of photorespiratory (*F*) and respiratory (*R*_d_) CO_2_ release to net CO_2_ uptake (*A*): *g*_m,app_ = *g*_m,dif_/[1 + *ω*(*F* + *R*_d_)/*A*], where *ω* is the fraction of *r*_ch_ in *r*_m,dif_. We herein extend the framework further by considering various scenarios for the intracellular arrangement of chloroplasts and mitochondria. We show that the formula of Tholen et al. implies either that mitochondria, where (photo)respired CO_2_ is released, locate between the plasmalemma and the chloroplast continuum or that CO_2_ in the cytosol is completely mixed. However, the model of Tholen et al. is still valid if *ω* is replaced by *ω*(1−*σ*), where *σ* is the fraction of (photo)respired CO_2_ that experiences *r*_ch_ (in addition to *r*_wp_ and stomatal resistance) if this CO_2_ is to escape from being refixed. Therefore, responses of *g*_m,app_ to (*F* + *R*_d_)/*A* lie somewhere between no sensitivity in the classical method (*σ* =1) and high sensitivity in the model of Tholen et al. (*σ* =0).

## Introduction

The biochemical C_3_ photosynthesis model of Farquhar, von Caemmerer and Berry (1980), the FvCB model hereafter, has been widely used to interpret leaf physiology from gas exchange measurements. The model calculates the net rate of leaf photosynthesis (*A*) as the minimum of the Rubisco carboxylation activity-limited rate (*A*_c_) and the electron (*e* ^−^) transport-limited rate (*A*_j_) of photosynthesis (see Appendix A). The partial pressure of CO_2_ at the carboxylation sites of Rubisco in the chloroplast stroma (*C*_c_) is a required input variable to calculate both *A*_c_ and *A*_j_ in the model. The drawdown of *C*_c_, relative to the CO_2_ level in the ambient air (*C*_a_), depends not only on stomatal conductance for CO_2_ transfer (*g*_sc_), but also on the mesophyll conductance for CO_2_ transfer between substomatal cavities and the site of CO_2_ carboxylation (*g*_m_). According to Fick’s diffusion law, *g*_m_ can be expressed as follows (von Caemmerer and Evans 1991; von Caemmerer et al. 1994):

1 $${{g}_{\text{m}}}=A/({{C}_{\text{i}}}-{{C}_{\text{c}}})$$

where *C*_i_ is the partial pressure of CO_2_ at the intercellular air spaces.

This simple gas diffusion equation has been combined with the FvCB model to estimate *g*_m_ (Pons et al. 2009), based on combined data of *A*–*C*_i_ curves and chlorophyll fluorescence measurements on photosystem II *e* ^−^ transport efficiency ${{\Phi }_{2}}$ (Harley et al. 1992; Yin and Struik 2009) or on combined gas exchange and carbon isotope discrimination measurements (Evans et al. 1986). When the *g*_m_ estimation is based on combined gas exchange and chlorophyll fluorescence measurements (e.g. the ‘variable J method’, Harley et al. 1992), the *A*_j_ part of the FvCB model is used, in which the linear *e* ^−^ transport rate (*J*) is estimated from chlorophyll fluorescence signals. Using this method, it has been reported that *g*_m_ can decrease with increasing *C*_i_ or with decreasing incoming irradiance *I*_inc_ (Flexas et al. 2007; Vrábl et al. 2009; Yin et al. 2009). Similar patterns of variable *g*_m_ have been reported with the isotope discrimination method (Vrábl et al. 2009), although with less consistency (Tazoe et al. 2009).

Equation (1) is based on net photosynthesis and assumes that respiratory and photorespiratory CO_2_ release occurs in the same compartment as CO_2_ fixation by Rubisco. However, CO_2_ fixation occurs in the chloroplast stroma, whereas (photo)respiratory CO_2_ is released in the mitochondria. The first step of photorespiration, the O_2_ fixation, takes place in the chloroplast to form phosphoglycolate. Phosphoglycolate is converted to glycolate and glyoxylate, and then to glycine in the peroxisome; glycine moves to the mitochondria and is decarboxylated there into CO_2_, NH_3_ and serine (Kebeish et al. 2007). The CO_2_ released in mitochondria, from either respiration or photorespiration, can be partially refixed by Rubisco in the chloroplast stroma, whereas the remaining portion escapes to the atmosphere (Busch et al. 2013). To quantify mesophyll resistance *r*_m_ (the reciprocal of *g*_m_), there is a need to specify resistance components within the cell imposed by walls, plasmalemma, cytosol, chloroplast envelope and stroma (Evans et al. 2009; Terashima et al. 2011). Unlike the CO_2_ that comes from the substomatal cavities, the CO_2_ from the mitochondria does not need to cross the cell wall and plasmalemma, and thus experiences a different resistance. Considering this difference, Tholen et al. (2012) developed a theoretical framework to analyse *g*_m_ as described below.

The total mesophyll diffusional resistance (*r*_m,dif_) can be described as the sum of a series of physical resistances comprising of intercellular air space, cell wall, plasmalemma, cytosol, chloroplast envelope and chloroplast stroma components (Evans et al. 2009): *r*_m,dif_ = *r*_ias_ + *r*_wall_ + *r*_plasmalemma_ + *r*_cytosol_ + *r*_envelope_ + *r*_stroma_. The resistance imposed by the gas phase component and the cytosol is generally small (Tholen et al. 2012), and may therefore be ignored. Tholen et al. (2012) combined *r*_wall_ and *r*_plasmalemma_ into the resistance at the cell wall–plasma membrane interface (*r*_wp_), and *r*_envelope_ and *r*_stroma_ into the total chloroplast resistance (*r*_ch_), so that *r*_m,dif_ = *r*_wp_ + *r*_ch_. Based on Fick’s diffusion law and considering two different resistance components encountered by CO_2_ from substomatal cavities and CO_2_ from the mitochondria, Tholen et al. (2012) derived the following relationship (their Eq. 6):

2 $${{C}_{\text{c}}}=~{{C}_{\text{i}}}-A\left( {{r}_{\text{wp}}}+{{r}_{\text{ch}}} \right)-(F+{{R}_{\text{d}}}){{r}_{\text{ch}}}$$

where *F* is the photorespiratory CO_2_ release and *R*_d_ is the CO_2_ release in the light other than by photorespiration, both in the mitochondria. The model Eq. (2) is still a simplification of true resistance pathways, because (i) diffusion is a continuous process and there are many parallel pathways (Tholen et al. 2012) and (ii) the model ignores that some respiratory flux originates in the chloroplast (Tcherkez et al. 2012) and that there may be small activity of phospho*enol*pyruvate carboxylase in cytosol (Douthe et al. 2012; Tholen et al. 2012).

Here we let *r*_ch_ = *ωr*_m,dif_; then *r*_wp_ = (1–*ω*)*r*_m,dif_, where *ω* is the relative contribution of *r*_ch_ to the total mesophyll resistance *r*_m,dif_ (= *r*_wp_+*r*_ch_). Equation (2) then becomes

3 $${{C}_{\text{c}}}=~{{C}_{\text{i}}}-A{{r}_{\text{m},\text{dif}}}-\omega (F+{{R}_{\text{d}}}){{r}_{\text{m},\text{dif}}}$$

Solving (*C*_i_−*C*_c_) from Eq. (3) and substituting it into Eq. (1) give

4 $${{g}_{\text{m}}}=\frac{1}{{{r}_{\text{m},\text{dif }\!\!~\!\!\text{ }}}\left( 1+\omega \frac{F+{{R}_{\text{d}}}}{A} \right)}$$

Equation (4) is equivalent to Eq. (9) of Tholen et al. (2012), in which *g*_wp_ and *g*_ch_ (i.e. the inverse of *r*_wp_ and *r*_ch_, respectively) are used. We prefer Eq. (4) because it allows (i) to analyse how *g*_m_ varies for a given total mesophyll resistance and (ii) to provide an analogue to an extended model that will be developed later.

Both Eq. (4) and Tholen et al.’s Eq. (9) tell that *g*_m_, as defined by Eq. (1), is influenced by the ratio of (photo)respiratory CO_2_ from the mitochondria to net CO_2_ uptake (*F* + *R*_d_)/*A*, thereby resulting in an apparent sensitivity of *g*_m_ to CO_2_ and O_2_ levels (Tholen et al. 2012). This sensitivity does not imply a change in the intrinsic diffusion properties of the mesophyll; so, *g*_m_ as defined by Eqs. (1) and (4) is apparent, and we denote it as *g*_m,app_ hereafter. The sensitivity depends on *ω*: the higher is *ω* the more sensitive is *g*_m,app_ to (*F* + *R*_d_)/*A*. If *ω* = 0, then *g*_m,app_ is no longer sensitive to (*F* + *R*_d_)/*A*, Eq. (3) becomes Eq. (1) and *g*_m,app_ becomes *g*_m,dif_—the intrinsic mesophyll diffusion conductance (= 1/*r*_m,dif_). In such a case, carboxylation and (photo)respiratory CO_2_ release occur in the same organelle compartment or if occurring in separate compartments, the chloroplast exerts a negligible resistance to CO_2_ transfer.

Equations (1) and (2) have been considered as two basic scenarios for CO_2_ diffusion path in C_3_ leaves (von Caemmerer 2013), both representing a simplified view on CO_2_ diffusion in the framework of whole leaf resistance models. Detailed views on the mechanistic basis of CO_2_ diffusion in relation to intracellular organelle positions could best be investigated using reaction–diffusion models (e.g. Tholen and Zhu 2011). However, uncertainties in the value of many required input diffusion coefficients and the complexity in nature are the major limitations of using these reaction–diffusion models (see Berghuijs et al. 2016 for discussions on simple resistance vs. reaction–diffusion models). We herein discuss an extended, yet simple, resistance model by considering various scenarios with regard to intracellular arrangement of organelles: (1) the relative positions of mitochondria and chloroplasts and (2) gaps between individual chloroplasts. We also discuss implications of these scenarios in estimating the fraction of (photo)respired CO_2_ being refixed.

## A generalised model

**Fig. 1:**
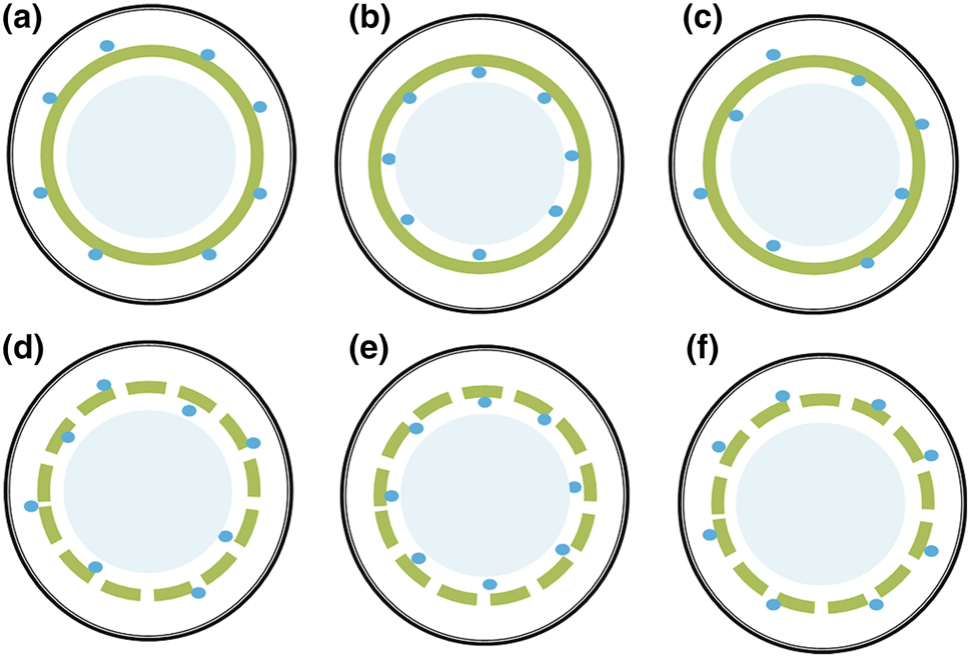
Schematic illustration of six scenarios for the arrangement of organelles in the mesophyll cell. In each panel, the *outer double-lined black circle* indicates the combined cell wall and plasmalemma, the *green circle* indicates chloroplast continuum (panels a–c) or the *green circle* segments indicate chloroplasts (panels d–f), the filled small *blue symbols* indicate mitochondria and the *inner light blue circle* represents vacuole

To develop a generalised model, we consider two possibilities of chloroplast distribution (either continuous or discontinuous) and three possibilities of mitochondria location (outer, inner or both outer and inner layers of cytosol). This gives six cases with regard to the arrangement of organelles within mesophyll cells (Fig. 1). In each scenario, mitochondria are intimately associated with chloroplasts, as commonly observed for real leaves (Sage and Sage 2009; Hatakeyama and Ueno 2016). Within our simple generalised model, we stay with the same notation of *r*_wp_ and *r*_ch_, the two-resistance components as the essence of the model of Tholen et al. (2012). However, as we discuss later on, instead of assuming that *r*_cytosol_ is negligible, we followed the approach of Berghuijs et al. (2015) that lumps part of *r*_cytosol_ into *r*_wp_ and the remaining part of *r*_cytosol_ into *r*_ch_. Given the position of mitochondria shown in Fig. 1, nearly all cytosolic resistance, i.e. along the diffusion path length from plasmalemma to chloroplast outer membrane, can be lumped into *r*_wp,_ whereas only a small remaining portion of *r*_cytosol_ is lumped into *r*_ch_.

### Case I

In this case, the coverage of chloroplasts is continuous and all mitochondria locate in the outer layer of cytosol (Fig. 1a). For this case, the net CO_2_ influx (*A*) from the intercellular air spaces is driven by the gradient between *C*_i_ and *C*_m(outer)_ (where *C*_m(outer)_ is the CO_2_ partial pressure at the outer layer of the mesophyll cytosol facing chloroplast envelope), whereas the gradient between *C*_m(outer)_ and *C*_c_ drives the carboxylation flux (*V*_c_). Therefore, equations for the CO_2_ gradient between the compartments and involved resistance components are as follows: *C*_c_ = *C*_m(outer)_−*V*_c_ *r*_ch_ and *C*_m(outer)_ = *C*_i_−*Ar*_wp_. In the FvCB model, *A* is formulated as *A* = *V*_c_−*F*−*R*_d_. Combining these three equations actually gives rise to Eq. (2), from which Eq. (4) for the sensitivity of *g*_m,app_ to (*F* + *R*_d_)/*A* was derived. Therefore, formulae for this Case I are in line with the framework as described by Tholen et al. (2012).

Tholen et al. (2012) also showed, based on their model framework, that the fraction of (photo)respired CO_2_ that is refixed by Rubisco can be quantified using the resistance components. We use *x*(*F* + *R*_d_) to denote the partial pressure of (photo)respired CO_2_ in mesophyll cytosol, where *x* is a conversion factor from flux to partial pressure for (photo)respired CO_2_ and has a unit of bar (mol m^− 2^ s^− 1^)^−1^. CO_2_ molecules from (photo)respiration can diffuse towards Rubisco but will experience *r*_ch_ and a resistance derived from the carboxylation itself (*r*_cx_); so the refixation rate (*R*_refix_) is *x*(*F* + *R*_d_)/(*r*_ch_+*r*_cx_). A portion of the (photo)respired CO_2_ molecules can also escape from refixation and move out of the stomata to the atmosphere, experiencing *r*_wp_ and the stomatal resistance for CO_2_ transfer *r*_sc_ (including a small boundary layer resistance); so the rate of this leak or escape (*R*_escape_) is *x*(*F* + *R*_d_)/(*r*_wp_+*r*_sc_). The fraction of (photo)respired CO_2_ that is refixed by Rubisco (*f*_refix_) can be calculated by

5 $${{f}_{\text{refix}}}=\frac{{{R}_{\text{refix}}}}{{{R}_{\text{refix}}}+{{R}_{\text{escape}}}}=\frac{\frac{1}{{{r}_{\text{ch}}}+{{r}_{\text{cx}}}}}{\frac{1}{{{r}_{\text{ch}}}+{{r}_{\text{cx}}}}+\frac{1}{{{r}_{\text{wp}}}+{{r}_{\text{sc}}}}}=\frac{{{r}_{\text{sc}}}+{{r}_{\text{wp}}}}{{{r}_{\text{sc}}}+{{r}_{\text{wp}}}+{{r}_{\text{ch}}}+{{r}_{\text{cx}}}}$$

This compares with Eq. (14) of Tholen et al. (2012) and shows that the refixation fraction can be calculated simply as the ratio of the resistance components that the escaped (photo)respired CO_2_ molecules have experienced to the total resistance along the full diffusion pathway.

### Case II

The coverage of chloroplasts is continuous and all mitochondria locate in the inner layer of cytosol, closely behind chloroplasts (Fig. 1b). In this case, since there are no mitochondria between the plasmalemma and chloroplasts, in essence, *r*_ch_ and *r*_wp_ can be combined and the flux involved is the same for the CO_2_ gradient between *C*_i_ and *C*_m(outer)_ and between *C*_m(outer)_ and *C*_c_, i.e. *A* (=*V*_c_–*F*–*R*_d_). This corresponds to the classical model, Eq. (1), that has commonly been used for estimating *g*_m_ (von Caemmerer and Evans 1991; von Caemmerer et al. 1994).

In this case, all (photo)respired CO_2_ molecules have to experience *r*_ch_, in addition to *r*_wp_ and *r*_sc_, if they are to escape from being refixed. As mitochondria locate closely behind chloroplasts and mitochondria and chloroplasts are treated essentially as one compartment in the classical model, (photo)respired CO_2_ molecules that diffuse towards Rubisco can be considered to experience *r*_cx_ only; so *R*_refix_ is *x*(*F* + *R*_d_)/*r*_cx_. The remaining (photo)respired CO_2_ that escape from refixation experience *r*_ch_, *r*_wp_ and *r*_sc_; so, *R*_escape_ is *x*(*F* + *R*_d_)/(*r*_ch_ + *r*_wp_+ *r*_sc_). Then, *f*_refix_ can be calculated by

6 $${{f}_{\text{refix}}}=\frac{{{R}_{\text{refix}}}}{{{R}_{\text{refix}}}+{{R}_{\text{escape}}}}=\frac{\frac{1}{{{r}_{\text{cx}}}}}{\frac{1}{{{r}_{\text{cx}}}}+\frac{1}{{{r}_{\text{ch}}}+{{r}_{\text{wp}}}+{{r}_{\text{sc}}}}}=\frac{{{r}_{\text{sc}}}+{{r}_{\text{wp}}}+{{r}_{\text{ch}}}}{{{r}_{\text{sc}}}+{{r}_{\text{wp}}}+{{r}_{\text{ch}}}+{{r}_{\text{cx}}}}$$

Obviously, this predicts a higher refixation fraction than Eq. (5) does.

### Case III

The coverage of chloroplasts is continuous and mitochondria locate in both inner and outer layers of cytosol (Fig. 1c). Let *λ* be the fraction of mitochondria that locate closely behind chloroplasts in the inner cytosol. Then (1−*λ*) is the fraction of mitochondria that locate in the outer cytosol. The flux associated with the gradient between *C*_m(outer)_ and *C*_c_ is the carboxylation flux (*V*_c_) minus the efflux of (photo)respired CO_2_ from the inner layer *λ* (*F* + *R*_d_), while the flux associated with the gradient between *C*_i_ and *C*_m(outer)_ is still *A*. Therefore, equations for the CO_2_ gradients between the compartments and involved resistance components are as follows:

7 $${{C}_{\text{c}}}=\text{ }\!\!~\!\!\text{ }{{C}_{\text{m}(\text{outer})}}-[{{V}_{\text{c}}}-\lambda \left( F+{{R}_{\text{d}}} \right)]{{r}_{\text{ch}}}$$

8 $$~{{C}_{\text{m}(\text{outer})}}={{C}_{\text{i}}}-A{{r}_{\text{wp}}}$$

Equation (7) without *R*_d_ would be comparable to the third equation in Fig. 4 of von Caemmerer (2013) for modelling the photorespiratory bypass engineered by Kebeish et al. (2007). Combining Eqs. (7) and (8) with *V*_c_ = *A* + *F* + *R*_d_ gives rise to an equation in analogy to Eq. (2):

9 $${{C}_{\text{c}}}=\text{ }\!\!~\!\!\text{ }{{C}_{\text{i}}}-A\left( {{r}_{\text{wp}}}+{{r}_{\text{ch}}} \right)-(1-\lambda )(F+{{R}_{\text{d}}}){{r}_{\text{ch}}}$$

The same logic as for Eqs. (3) and (4) gives

10 $${{g}_{\text{m},\text{app}}}=\frac{1}{{{r}_{\text{m},\text{dif }\!\!~\!\!\text{ }}}\left[ 1+\omega (1-\lambda )\frac{F+{{R}_{\text{d}}}}{A} \right]}$$

Equation (10) suggests that the apparent *g*_m_ as defined by Eq. (1) is still sensitive to (*F* + *R*_d_)/*A*, although the sensitivity factor changes from *ω* for Case I to *ω*(1−*λ*) now for Case III.

For this case, either refixed or escaped (photo)respired CO_2_ molecules have two parts, one part from the inner and the other from outer cytosol, and they experience different resistant components. Assuming for the purpose of simplicity that mitochondria are distributed in such a way that any variation in *x* between inner and outer cytosol is negligible, the refixed (photo)respired CO_2_ molecules *R*_refix_ can easily be expressed as *λx*(*F* + *R*_d_)/*r*_cx_ + (1−*λ*)*x*(*F* + *R*_d_)/(*r*_ch_ + *r*_cx_), whereas the escaped (photo)respired CO_2_ *R*_escape_ can be expressed as *λx*(*F* + *R*_d_)/(*r*_ch_ + *r*_wp_ + *r*_sc_) + (1−*λ*)*x*(*F* + *R*_d_)/(*r*_wp_+*r*_sc_). Then, *f*_refix_ can be calculated by

11 $${{f}_{\text{refix}}}=\frac{{{R}_{\text{refix}}}}{{{R}_{\text{refix}}}+{{R}_{\text{escape}}}}=\frac{\frac{\lambda }{{{r}_{\text{cx}}}}+\frac{1-\lambda }{{{r}_{\text{ch}}}+{{r}_{\text{cx}}}}}{\frac{\lambda }{{{r}_{\text{cx}}}}+\frac{1-\lambda }{{{r}_{\text{ch}}}+{{r}_{\text{cx}}}}+\frac{\lambda }{{{r}_{\text{ch}}}+{{r}_{\text{wp}}}+{{r}_{\text{sc}}}}+\frac{1-\lambda }{{{r}_{\text{wp}}}+{{r}_{\text{sc}}}}}$$

This expression for *f*_refix_ looks rather unwieldy but it covers Eqs. (5) and (6) for the previous two cases when *λ* is 0 and 1, respectively.

### Case IV

This is the most general case, in which the coverage of chloroplasts is discontinuous and mitochondria locate in both inner and outer layers of cytosol (Fig. 1d). If chloroplast coverage is discontinuous, it is possible that some mitochondria lie exactly in the chloroplast gaps. This situation can be simplified by assigning part of (photo)respired CO_2_ in the gaps to the inner and the other part to the outer cytosol; so, *λ* is still defined as for Case III as the fraction of mitochondria that locate in the inner cytosol. However, another factor needs to be introduced to account for the direct effect of the chloroplast gaps as these gaps allow the diffusion of (photo)respired CO_2_ from the inner to the outer cytosol and vice versa. In our context here, we only need to define *k* as the factor allowing for a decrease (0 ≤ *k* < 1) or an increase (*k* > 1) in the fraction of inner (photo)respired CO_2_, caused by the chloroplast gaps. Then, Eq. (7) can be simply adjusted for Case IV:

12 $${{C}_{\text{c}}}=~{{C}_{\text{m}(\text{outer})}}-[{{V}_{\text{c}}}-k\lambda \left( F+{{R}_{\text{d}}} \right)]{{r}_{\text{ch}}}$$

while Eq. (8) remains unchanged. Then, equations for case IV, equivalent to Eqs. (9–11) for case III, can be easily defined by replacing the places of *λ* with *kλ*. This also means that the fraction of outer (photo)respired CO_2_ now becomes (1−*kλ*).

In fact, the lumped *kλ* can be re-defined as a single factor *σ*, which refers to the fraction of (photo)respired CO_2_ molecules that have to experience *r*_ch_, in addition to *r*_wp_ and *r*_sc_, if they are to escape from being refixed. Then, a more general form of Eq. (3) or Eq. (9) becomes

13 $${{C}_{\text{c}}}=~{{C}_{\text{i}}}-A{{r}_{\text{m},\text{dif}}}-\omega (1-\sigma )(F+{{R}_{\text{d}}}){{r}_{\text{m},\text{dif}}}$$

and a more general form of Eqs. (10) and (11) becomes

14 $${{g}_{\text{m},\text{app}}}=\frac{1}{{{r}_{\text{m},\text{dif }\!\!~\!\!\text{ }}}\left[ 1+\omega (1-\sigma )\frac{F+{{R}_{\text{d}}}}{A} \right]}$$

15 $${{f}_{\text{refix}}}=\frac{{{R}_{\text{refix}}}}{{{R}_{\text{refix}}}+{{R}_{\text{escape}}}}=\frac{\frac{\sigma }{{{r}_{\text{cx}}}}+\frac{1-\sigma }{{{r}_{\text{ch}}}+{{r}_{\text{cx}}}}}{\frac{\sigma }{{{r}_{\text{cx}}}}+\frac{1-\sigma }{{{r}_{\text{ch}}}+{{r}_{\text{cx}}}}+\frac{\sigma }{{{r}_{\text{ch}}}+{{r}_{\text{wp}}}+{{r}_{\text{sc}}}}+\frac{1-\sigma }{{{r}_{\text{wp}}}+{{r}_{\text{sc}}}}}$$

As *σ* has a value between 0 and 1, it follows that the factor *k* varies between 0 and 1/*λ*. This suggests that the lower the *λ* is, the more likely it is that *k* > 1. However, the exact value of *k* and how *k* modifies *λ* (e.g. via the path between the chloroplasts vs through the chloroplast) are hard to quantify from the simple resistance model. As large gaps between chloroplasts decrease *S*_c_/*S*_m_, the ratio of chloroplast surface area to mesophyll surface area exposed to the intercellular air spaces (Sage and Sage 2009; Tholen et al. 2012; Tomas et al. 2013), the value of *k* must be associated with *S*_c_/*S*_m_. However, *k* may also depend on factors such as the CO_2_ influx from the intercellular air spaces. These dependences of *k* on *λ, S*_c_/*S*_m_, and other factors could best be analysed using reaction–diffusion models like the one by Tholen and Zhu (2011).

### Two more special cases

Now we consider two more special cases. The first instance is the case in which the coverage of chloroplasts is discontinuous and all mitochondria locate in the inner layer of cytosol (Fig. 1e), and the second is that the coverage of chloroplasts is discontinuous and all mitochondria locate in the outer layer of cytosol (Fig. 1f). The diffused amount of (photo)respired CO_2_ from the inner to the outer cytosol (for the first instance) or from the outer to the inner cytosol (for the second instance) could be analysed by the use of a reaction–diffusion model. Again if* σ* also refers to the fraction of (photo)respired CO_2_ molecules that have to experience *r*_ch_, in addition to *r*_wp_ and *r*_sc_, if they are to escape from being refixed, Eqs. (13–15) also apply to these two special cases.

## Results and discussion

### Dependence of *A* and *g*_m,app_ on *ω* and *σ* values

**Fig. 2:**
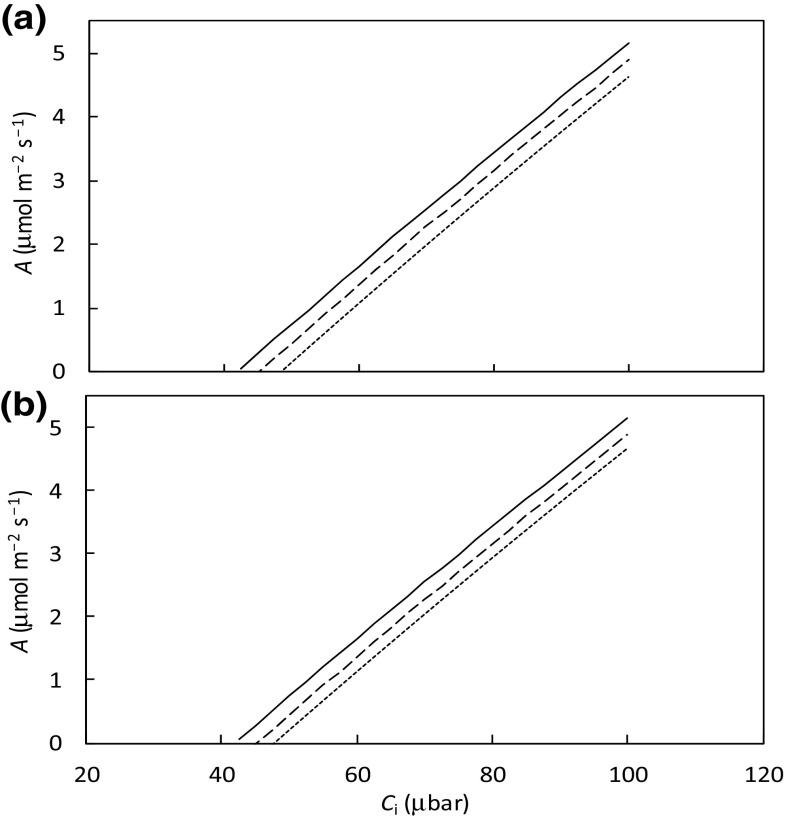
Simulated net CO_2_ assimilation rate (*A*) as a function of low *C*_i_, under ambient O_2_ condition: **a** for three values of *σ* (*solid line* for *σ* = 1, *long-dashed line* for *σ* = 0.5 and *short*-dashed *line* for *σ* = 0) if parameter *ω* stays constant at 0.5, and **b** for three values of *ω* (*solid line* for *ω* = 0, *long*-dashed *line* for *ω* = 0.5 and *short*-dashed *line* for *ω* = 0.9) if parameter *σ* stays constant at 0.5. Other parameter values used for this simulation: *g*_m,dif_ = 0.4 mol m^− 2^ s^− 1^ bar ^− 1^; *V*_cmax_ = 80 μmol m^− 2^ s^− 1^; *K*_mC_ = 291 μbar; *K*_mO_ = 194 mbar; *R*_d_ = 1 μmol m^− 2^ s^− 1^ and Rubisco specificity *S*_c/o_ = 3.1 mbar μbar^− 1^ (the equivalent $\Gamma$_*_ = 34 μbar for the ambient O_2_ condition). Simulation used Eqn (18) in Appendix A

Equations for all illustrations in this section are all given in Appendix A. Figure 2 shows the initial section of simulated *A*–*C*_i_ curves for various combinations of *ω* and *σ* values, indicating that a change in *σ* (i.e. the arrangement of chloroplasts and mitochondria in mesophyll cells) had a same magnitude of the effect as a change in *ω* (i.e. the physical resistance of chloroplast components relative to the total mesophyll resistance). Increasing *σ* (Fig. 2a) or decreasing *ω* (Fig. 2b) increased *A* for a given *g*_m,dif_. This is largely caused by varying amounts of refixation of (photo)respired CO_2_, which become increasingly important with decreasing *C*_i_. For example, the estimated *f*_refix_ (Eq. 15) was 0.385, 0.333 and 0.285 for the three cases corresponding to solid, long-dashed and short-dashed lines of Fig. 2a, respectively (where *r*_sc_ was set to have the same value as 1/*g*_m,dif_, and *r*_cx_ was calculated as (*C*_c_ + *x*_2_)/*x*_1_, also see Eq. B2 in Tholen et al. 2012). *f*_refix_ can also be calculated for the three cases of Fig. 2b. Such differences in *f*_refix_ can produce a significant difference in *A* (when *C*_i_ is low) and in CO_2_ compensation point $\Gamma$ (Fig. 2). Differences in $\Gamma$ was already shown by von Caemmerer (2013) between two special cases, i.e. Case I (Fig. 1a) versus Case II (Fig. 1b). With increasing *C*_i_, refixation becomes less important, and differences in *A* are increasingly negligible (results not shown).

**Fig. 3:**
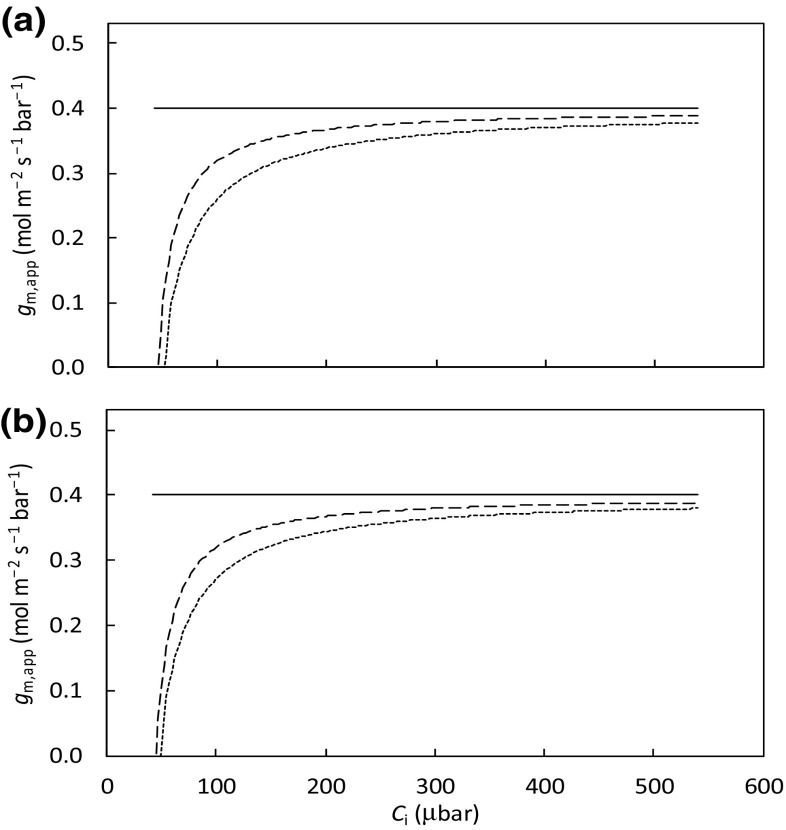
Simulated apparent mesophyll conductance (*g*_m,app_) as a function of *C*_i_, under ambient O_2_ condition: **a** for three values of *σ* (*solid line* for *σ* = 1, *long-dashed line* for *σ* = 0.5 and *short dash line* for *σ* = 0) if parameter *ω* stays constant at 0.5 and **b** for three values of *ω* (*solid line* for *ω* = 0, *long-dashed line* for *ω* = 0.5 and *short-dashed line* for *ω* = 0.9) if parameter *σ* stays constant at 0.5. The value of *J* used for simulation was 125 μmol m^− 2^ s^− 1^. Other parameter values as in Fig. 2. Simulation used the method as described in Appendix A

*g*_m,app_, calculated from Eq. (14), decreased with decreasing *C*_i_, although *g*_m,dif_ was fixed as constant (Fig. 3). This variation did not occur only if *σ* = 1 (the horizontal line in Fig. 3a) or *ω* = 0 (the horizontal line in Fig. 3b), suggesting the classical *g*_m_ model can arise either from *σ* = 1 (all mitochondria stay closely behind chloroplasts as if carboxylation and (photo)respiratory CO_2_ release occur in one compartment) or from *ω* = 0 (the chloroplast component in total mesophyll resistance is negligible). The short-dashed line in Fig. 3a represents the case when *σ* = 0, corresponding to the original model of Tholen et al. (2012) that applies to the case where all mitochondria locate in the outer cytosol. A change in organelle arrangement within a mesophyll cell resulted in a change in sensitivity of *g*_m,app_ to *C*_i_ as shown by the long-dashed line in Fig. 3a, which lies between the horizontal line and the short-dashed line.

### The model of Tholen et al. (2012) as special case of the generalised model

It is evident from our analysis above that the original model of Tholen et al. (2012) applies to a special case of our generalised model, where (photo)respired CO_2_ is entirely released in the outer cytosol between the plasmalemma and the chloroplast layer. However, this case can hardly be observed in real leaves, where mitochondria occur mostly in the cell interior, closely behind chloroplasts (Sage and Sage 2009; Hatakeyama and Ueno 2016).

In our model, as stated earlier for the purpose of retaining model simplicity, a large part of *r*_cytosol_ is lumped into *r*_wp_, and the remaining part is lumped into *r*_ch_. For their model, Tholen et al. (2012) assumed that cytosolic resistance is negligible. Although this assumption was made, as described by Tholen et al. (2012), only for the purpose of simplicity, it has implications. If *r*_cytosol_ is so small that it can be neglected, then CO_2_ diffusion is so fast that the CO_2_ concentration anywhere in the cytosol should be the same independent of where the mitochondria are located, provided the cytosol is continuous (for example, allowed by an *S*_c_/*S*_m_ lower than 1). Then the position of the mitochondria does not have any effect on *f*_refix_. Practically, the four cases for scenarios (a), (d), (e) and (f) in Fig. 1 would all be equivalent to the original Tholen et al. model (*σ*  = 0). This is because *λ* = 0 in the case of Fig. 1a, or *k* = 0 in cases of Fig. 1d,e, or both *λ* and *k* = 0 in the case of Fig. 1 f. In this context, the original model of Tholen et al. (2012) would become an alternative special case of our model, that is, assuming that CO_2_ in the cytosol is completely mixed. If *r*_cytosol_ is indeed negligible, then cases in Fig. 1d,e,f are no longer needed for developing the generalised model.

### Can parameters *ω* and *σ* in the generalised model be measured?

In real cells, *r*_cytosol_ may be very high (Peguero-Pina et al. 2012; Berghuijs et al. 2015) and therefore cannot be neglected. Then, *r*_cytosol_ should appear in the model, making it dependent on the detailed morphology of the cell and location of mitochondria and chloroplasts, and this would require the use of a reaction–diffusion model. Within the resistance model framework, Tholen et al. (2012, in their Appendix C) and Tomas et al. (2013) analysed the possible effects of *r*_cytosol_ in relation to *S*_c_/*S*_m_ on *g*_m_. In our generalised model, any significant *r*_cytosol_ value would mainly be lumped into parameter *ω*, while parameter *σ* encompasses any combination of chloroplast–mitochondria arrangement and *S*_c_/*S*_m_. This means that parameters *ω* and *σ* in our model can be experimentally measured, at least approximately.

Individual physical resistance components *r*_wall_, *r*_plasmalemma_, *r*_cytosol_, *r*_envelope_ and *r*_stroma_ have been calculated from microscopic measurements on leaf anatomy (Peguero-Pina et al. 2012; Tosens et al. 2012a, b; Tomas et al. 2013; Berghuijs et al. 2015), despite the uncertainties in the value of gas diffusion coefficients used for the calculation. These measurements can provide basic data to derive *ω*. For example, Berghuijs et al. (2015) showed that for tomato leaves, *ω* was about 0.65. Parameter *σ* depends on both *S*_c_/*S*_m_ and the relative position of mitochondria to chloroplasts. In most annuals especially when leaves are young, *S*_c_/*S*_m_ is high (close to 1; Sage and Sage 2009; Terashima et al. 2011; Berghuijs et al. 2015), *σ* should be predominantly determined by the relative position of mitochondria (i.e. *σ * ≈ *λ*, the proportion of mitochondria lying in the inner cytosol). Hatakeyama and Ueno (2016) showed that for 10 C_3_ grasses most mitochondria are located on the vacuole side of chloroplast in mesophyll cells and their data suggested that *λ* varies from 0.61 to 0.92 among these species, with an average of 0.8. Assuming these values are representative for young leaves of annual C_3_ species, then the collective value of *ω*(1−*σ*) in our model is about 0.13, a value closer to what the classical model represents (0) than the model of Tholen et al. (2012) does.

However, in woody species (e.g. Tosens et al. 2012a) or in old leaves of annual species (Busch et al. 2013), *S*_c_/*S*_m_ can be as low as 0.4. Because the chloroplast coverage is low, especially when combined with a low *r*_cytosol_ (Tosens et al. 2012b), *ω*(1−*σ*) must be close to what the model of Tholen et al. (2012) represents. However, parameter *σ* is hard to determine directly for this case as its component *k* may be interdependent on its other component *λ*. In such a case, *σ* may only be a “fudge factor” that lumps *λ* and *S*_c_/*S*_m_ in a complicated manner, which may be elucidated by using reaction–diffusion models. Alternatively, the collective value of *ω*(1−*σ*) could be estimated (together with *g*_m,dif_) by fitting Eq. (18) in AppendixA to gas exchange data at various O_2_ levels, and then *σ* could be calculated if anatomical measurements reliably estimate* ω*; but this approach needs to be tested.

### Can two-resistance models exclusively explain observed variable *g*_m,app_?

Compared with the classical model that uses a single resistance parameter, both Tholen et al. (2012) model and our generalised model partition mesophyll resistance into two components. In Fig. 3, we have shown the dependence in the sensitivity of *g*_m,app_ on both *ω* and *σ* values. Our illustration for the general case (Fig. 3) still agrees qualitatively with Tholen et al. (2012), who, based on their two-resistance model, clearly showed the sensitivity of *g*_m,app_ to the ratio of (*F* + *R*_d_) to *A*. They suggested that this sensitivity could explain the commonly observed decrease of *g*_m,app_ with decreasing *C*_i_ with a low *C*_i_ range (e.g. Flexas et al. 2007; Yin et al. 2009). Since the (*F* + *R*_d_)/*A* ratio also varies with irradiance and temperature, one might wonder if their model explains any variation of *g*_m,app_ with these factors. However, their framework, as stated by Tholen et al. (2012), cannot explain the commonly observed responses of *g*_m,app_ to a change in *C*_i_ within the higher *C*_i_ range (e.g. Flexas et al. 2007) or in *I*_inc_ (e.g. Yin et al. 2009; Douthe et al. 2012) or in temperature (e.g. Bernacchi et al. 2002; Yamori et al. 2006; Evans and von Caemmerer 2013; von Caemmerer and Evans 2015). In fact, Gu and Sun (2014) showed that even the response of *g*_m,app_ to a change in *C*_i_ (including the low *C*_i_ range) could be simply due to possible errors in measuring *A, J* and *C*_i_, or to possible errors in estimating *R*_d_ and *S*_c/o_, or could be due to the use of the NADPH-limited form of the FvCB model by the variable J method when the true form is the ATP-limited equation.

**Fig. 4:**
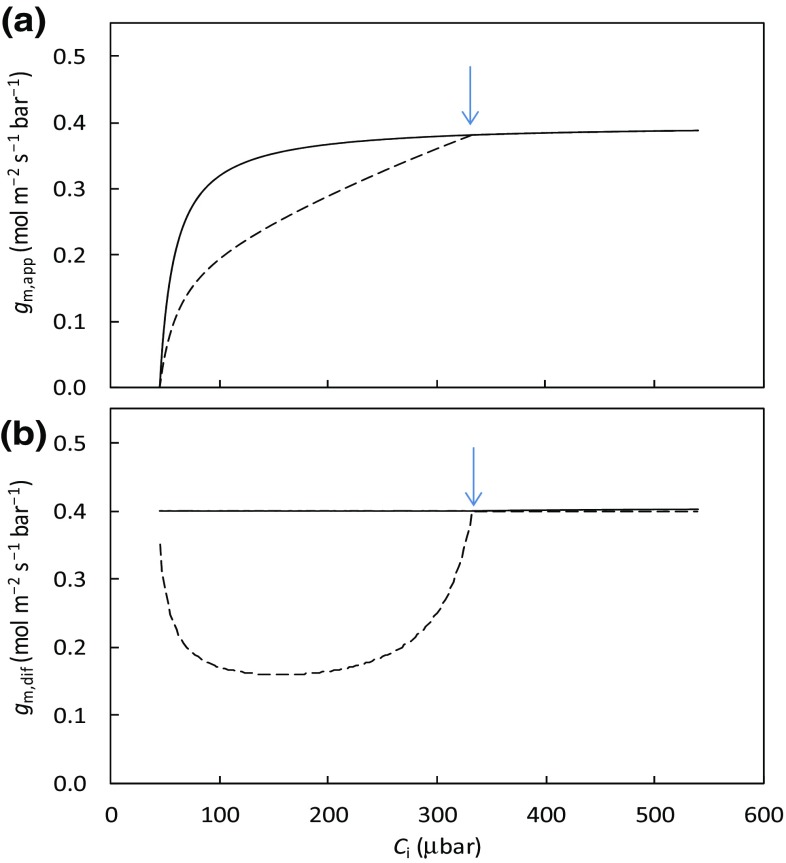
Simulated apparent mesophyll conductance *g*_m,app_ (**a**) or intrinsic diffusional mesophyll conductance *g*_m,dif_ (**b**) as a function of *C*_i_ under ambient O_2_ condition, with *σ* = 0.5 and *ω* = 0.5. *g*_m,app_ and *g*_m,dif_ were calculated using *C*_c_ derived either from the full FvCB model (*solid lines*) or from the *A*_j_ part of the model as in the variable J method (dashed *lines*). The *arrow* indicates the transition point from being *A*_c_-limited to being *A*_j_-limited. Input parameter values used for simulation are given in Figs. 2 and 3. Simulation used the method as described in Appendix A

In the absence of any measurement errors, can the sensitivity of *g*_m,app_ to the (*F* + *R*_d_)/*A* ratio be considered as the only explanation of *g*_m,app_ sensitivity to *C*_i_ within the low *C*_i_ range? Here we want to (re-)state that the decline of *g*_m,app_ with decreasing *C*_i_ below a certain level, as assessed by the variable J method of Harley et al. (1992), can also be accounted for by the fact that the method is based only on the *A*_j_ equation of the FvCB model (Yin et al. 2009). When *C*_i_ is decreasing towards the CO_2_ compensation point, *A* is increasingly limited by *A*_c_ rather than by *A*_j_. Under such conditions, part of the *e* ^−^ fluxes may become alternative *e* ^−^ transport not used in support of CO_2_ fixation and photorespiration. So, use of the variable J method, which is based on Eq. (1) and the *A*_j_ equation of the FvCB model, may lead to underestimation of *g*_m,app_. This is shown in Fig. 4a, in which for a given fixed *g*_m,dif_ (0.4 mol m^− 2^ s^− 1^ bar ^− 1^), *g*_m,app_ decreased with decreasing *C*_i_ as expected from Eq. (14); but *g*_m,app_ decreased more sharply if *A*_j_ part of the model was applied to the low *C*_i_ range which was actually *A*_c_-limited. One would expect that *g*_m,dif_ calculated back from using the simulated *A* should be equal to the pre-fixed *g*_m,dif_ (0.4 mol m^− 2^ s^− 1^ bar ^− 1^). However, the calculated *g*_m,dif_ if using only the *A*_j_ part of the model as in the variable J method gave artifactually lower *g*_m,dif_ values for the *A*_c_-limited part (Fig. 4b). In this calculation shown in Fig. 4, *J* was assumed to be a constant across *C*_i_ levels, whereas actual fluorescence measured *J* may decline slightly with lowering *C*_i_ in the low *C*_i_ range (e.g. Cheng et al. 2001), probably reflecting a feedback effect of Rubisco limitation on electron transport. However, the feedback is not so complete that the variable J method, if applied to the low *C*_i_ range, always tends to underestimate the actual mesophyll conductance. For these reasons, Yin and Struik (2009) stated that the proposal of the variable J method to be applied to the lower range of *A*–*C*_i_ curve where *J* is variable (Harley et al. 1992) is inappropriate. A good correlation between values of *g*_m_ estimated from the variable J method and the online isotopic method but not when *C*_i_ is <200 µmol mol^− 1^ (Vrábl et al. 2009) further supports our statement.

## Conclusions

The model of Tholen et al. (2012) considers the partitioning of intrinsic diffusion resistance but with little explicit consideration of intracellular organelle arrangements, especially not intracellular position of mitochondria and chloroplasts. We introduced the parameter *σ* for defining the fraction of (photo)respired CO_2_ molecules that have to experience all *r*_ch_, in addition to *r*_wp_ and *r*_sc_, if these CO_2_ molecules are to escape from being refixed. *σ* has a value between 0 and 1, depending on the arrangement of organelles within mesophyll cells, i.e. (1) the relative position of chloroplasts and mitochondria and (2) the size of the gaps between chloroplasts. This provides a simple generalised form of the Tholen et al. model in a way that the latter model, Eq. (4), is still valid for all organelle arrangement scenarios if *ω* is replaced by *ω*(1−*σ*). The two parameters of our generalised model can be amenable to experimental estimation for young leaves of annual species where chloroplast coverage continues along the mesophyll cell periphery (*S*_c_/*S*_m_ = 1). The model of Tholen et al. (2012) is the special case of our model when *σ* = 0, which arises either from *λ* = 0 (no mitochondria in the inner cytosol) combined with *S*_c_/*S*_m_ = 1 or from a negligible *r*_cytosol_ combined with *S*_c_/*S*_m_ < 1. Our model shows that the sensitivity of *g*_m,app_ to (*F* + *R*_d_)/*A* lies somewhere in between the classical method (*ω* = 0 or* σ* = 1, non-sensitive) and the Tholen et al. model (*σ* = 0, highly sensitive). Therefore, Tholen et al. (2012) may have overstated that the sensitivity of *g*_m,app_ on (*F* + *R*_d_)/*A* in their model explains the commonly reported decline of *g*_m,app_ with decreasing *C*_i_ in the low *C*_i_ range. In fact, the decline, if not due to measurement or parameter–estimation errors, could also be attributed, at least partly, to the variable J method that is wrongly applied to low *C*_i_ range where CO_2_ assimilation is actually limited by Rubisco activity.

## Appendix A

### Equations used for simulation in this paper

The FvCB model calculates carboxylation rate (*V*_c_) as *C*_c_ *x*_1_/(*C*_c_ + *x*_2_), and photorespiratory CO_2_ release *F* as ($\Gamma$_*_/*C*_c_)*V*_c_ ; so, *F* can be expressed as follows:

16 $$F=\frac{{{\Gamma }_{*}}{{x}_{1}}}{{{C}_{\text{c}}}+{{x}_{2}}}$$

where $\Gamma$_*_ is the CO_2_ compensation point in the absence of *R*_d_ ($\Gamma$_*_ depends on O_2_ partial pressure *O* and Rubisco specificity *S*_c/o_ as 0.5*O*/*S*_c/o_), *x*_1_ = *J*/4 and *x*_2_ = 2$\Gamma$_*_ for the *A*_j_-limited conditions and *x*_1_ = *V*_cmax_ (maximum carboxylation activity of Rubisco) and *x*_2_ = *K*_mC_(1 + *O*/*K*_mO_) for the *A*_c_-limited conditions (*K*_mC_ and *K*_mO_ are Michaelis–Menten constants of Rubisco for CO_2_ and O_2_, respectively).

Also *C*_c_ can be solved from the FvCB model as

17 $${{C}_{\text{c}}}=\frac{{{\Gamma }_{*}}{{x}_{1}}+{{x}_{2}}(A+{{R}_{\text{d}}})}{{{x}_{1}}-(A+{{R}_{\text{d}}})}$$

Combining Eqs. (16–17) with Eq. (13) and solving the combined equations for *A*, step by step, result in a quadratic solution:

18 $$A=(-b-\sqrt{{{b}^{2}}-4ac})/(2a)$$

where $$a={{x}_{2}}+{{\Gamma }_{*}}[1-\omega (1-\sigma )]$$

$$b=\omega \left( 1-\sigma \right)\left( {{R}_{\text{d}}}{{x}_{2}}+{{\Gamma }_{*}}{{x}_{1}} \right)-\left\{ {{x}_{2}}+{{\Gamma }_{*}}\left[ 1-\omega \left( 1-\sigma \right) \right] \right\}\left( {{x}_{1}}-{{R}_{\text{d}}} \right)-{{g}_{\text{m},\text{dif}}}\left( {{C}_{\text{i}}}+{{x}_{2}} \right)\left( {{x}_{2}}+{{\Gamma }_{*}} \right)$$

$$c=-\omega \left( 1-\sigma \right)\left( {{R}_{\text{d}}}{{x}_{2}}+{{\Gamma }_{*}}{{x}_{1}} \right)\left( {{x}_{1}}-{{R}_{\text{d}}} \right)+{{g}_{\text{m},\text{dif}}}\left( {{x}_{2}}+{{\Gamma }_{*}} \right)[{{x}_{1}}\left( {{C}_{\text{i}}}-{{\Gamma }_{*}} \right)-{{R}_{\text{d}}}\left( {{C}_{\text{i}}}+{{x}_{2}} \right)]$$

Equation (18) was used to generate *A*–*C*_i_ response curves as shown in Fig. 2 for a given set of input parameter values.

When *A* is solved, *C*_c_ can be solved from Eq. (18), and the solved *C*_c_ was used as input to Eq. (1) to calculate *g*_m,app_. Alternatively, *g*_m,app_ can be calculated directly from Eq. (14) with *F* calculated from eqn (16). This gave *g*_m,app_ as shown in Figs. 3 and 4a.

When *A, C*_c_ and *F* are all known, one can calculate back for *g*_m,dif_:

19 $${{g}_{\text{m},\text{dif}}}=\frac{A+\omega (1-\sigma )(F+{{R}_{\text{d}}})}{{{C}_{\text{i}}}-{{C}_{\text{c}}}}$$

When *C*_c_ and *F* are both based on the *A*_j_-limited part of the FvCB model, then eqn (19) gives an equation that calculates *g*_m,diff_, like the variable J method of Harley et al. (1992) for calculating *g*_m,app_. This was used to generate Fig. 4b.
